# Ion channel gene signature for diagnosis and antifibrotic therapy in liver fibrosis

**DOI:** 10.1186/s12967-026-07856-1

**Published:** 2026-02-16

**Authors:** Yun Li, Duoer Shen, Fusheng Qin, Dongkui Chen, Jianguo Li

**Affiliations:** 1 Department of Gastroenterology, The Fourth Hospital of Changsha, Changsha, 410006 Hunan China; 2 Department of Cardiology, The Fourth Hospital of Changsha, Changsha, 410006 Hunan China; 3 Department of Nephrology, The Fourth Hospital of Changsha, Changsha, 410006 Hunan China

**Keywords:** Liver fibrosis, Ion channel gene, GJA1, AQP1, KCNN2

## Abstract

**Background:**

Liver fibrosis (LF) is a progressive pathological process that may lead to cirrhosis and liver failure. Human ion channel genes (HICGs) participate in hepatic mechanotransduction and immune regulation, but their contributions to LF remain insufficiently characterized. This study aimed to profile the expression of HICGs in LF and to identify key genes with diagnostic and therapeutic relevance.

**Methods:**

Multiple transcriptomic datasets were integrated to identify differentially expressed HICGs in LF. Weighted gene co-expression network analysis and single-cell RNA sequencing were applied to identify fibrosis-associated gene modules and cell-type distribution. Functional enrichment and immune infiltration analyses were performed to explore biological relevance. The expression of key genes was validated in human cirrhotic tissues and bile duct ligation mouse models using immunohistochemistry. Potential therapeutic compounds targeting hub HICGs were predicted through molecular docking simulations.

**Results:**

Three HICGs—*AQP1*, *GJA1*, and *KCNN2*—were identified as fibrosis-associated hub genes, showing distinct expression patterns and high diagnostic performance. *GJA1* showed consistent upregulation in both experimental models and human cirrhosis. Functional analyses linked these genes to extracellular matrix remodeling, cell adhesion, and cytokine interactions, while immune infiltration analysis revealed significant associations with M0 macrophages, plasma cells, NK cells, and memory B cells. Molecular docking simulations further identified 16 candidate drugs targeting KCNN2 and GJA1.

**Conclusions:**

This study demonstrates that *AQP1*, *GJA1*, and *KCNN2* are closely associated with LF progression and immune remodeling. The consistent upregulation of *GJA1*, together with the identification of candidate drug interactions, provides potential avenues for biomarker development and therapeutic repurposing in LF.

**Supplementary Information:**

The online version contains supplementary material available at 10.1186/s12967-026-07856-1.

## Introduction

Liver fibrosis (LF) is a common pathological consequence of chronic liver injury, characterized by excessive deposition and abnormal remodeling of the extracellular matrix (ECM). If left unchecked, LF can progress to liver cirrhosis (LC), hepatic failure, and hepatocellular carcinoma, posing a significant global health burden [1]. Notably, even at the decompensated stage of cirrhosis, LF remains a dynamic process and is partially reversible [2, 3]. Therefore, elucidating its molecular mechanisms is critical for reversing fibrosis. Emerging evidence suggests that fibrosis is closely linked to biomechanical signaling, with ion channels playing essential roles.

Ion channels are membrane-embedded pore-forming proteins that regulate selective ion flux (e.g., Na⁺, K⁺, Ca²⁺) in response to voltage, ligands, mechanical force, pH, or temperature, thereby controlling gated and selective ion diffusion [4]. According to the HUGO Gene Nomenclature Committee, the human ion channel gene (HICG) family comprises 330 genes, including ion channels and ion transporters. Beyond their physiological roles in neuronal signaling, cardiac electrophysiology, and renal transport, HICGs are implicated in multiple disorders and represent therapeutic targets, such as in epilepsy, arrhythmia, gastrointestinal dysfunction and malignancies [5–9]. HICGs also critically contribute to organ fibrosis [10], particularly the mechanosensitive channel *Piezo1*. Macrophage-expressed *Piezo1* regulates cathepsin S transcription and activity through the calpain/LAMP1 pathway, thereby influencing LF [2]. Moreover, matrix stiffening activates *Piezo1* in macrophages, which enhances macrophage efferocytosis and prevents inflammation, contributing to fibrosis resolution [11]. Other HICGs have also been implicated in fibrosis regulation. For example, the capsaicin receptor TRPV1 is highly expressed in quiet hepatic stellate cells and recruits SARM1 to inhibit TRPV1-mediated calcium influx, thereby suppressing hepatic stellate cell activation and making it a potential therapeutic target for LF [12]. Furthermore, chloride channels (e.g., *TMEM16A*) and aquaporins (*AQPs*) contribute to fibrosis through the regulation of cell volume, ion transport, and inflammatory responses, both in the liver and other organs [13–15]. Despite significant progress in these studies, the overall expression profiles of HICGs in LF remain unknown, particularly at the transcriptome level, and the potential for HICGs to serve as diagnostic biomarkers or therapeutic targets in LF is underexplored.

In the present study, we performed comprehensive transcriptome profiling of HICGs in LF, integrating differential expression, co‑expression network analyses and validation in animal and human liver tissues. Our aim was to identify candidate ion‑channel‑associated biomarkers and explore their mechanistic links to fibrogenesis, thereby facilitating the identification of novel diagnostic markers and potential antifibrotic targets.

## Methods

### Transcriptomic data processing and identification of HICGs in LF

The 330 ion channel genes listed by the HUGO Gene Nomenclature Committee were utilized to identify relevant HICGs in LF (https://www.genenames.org/data/genegroup/#!/). Three publicly available transcriptomic datasets (GSE14323, GSE197112, and GSE84044) were randomly retrieved from the GEO database [16]. These datasets were selected based on the following criteria: (1) containing liver tissue samples from both patients with LF and non-fibrotic controls; (2) gene expression profiling was achieved by array; and (3) availability of raw or normalized expression data. Detailed characteristics of the datasets, including etiology of LF, are summarized in Supplementary Table 1. To assess the batch effects across datasets, we performed principal component analysis. Data normalization and batch effect correction were subsequently conducted using the limma (v3.60.6) and sva (v3.52.0) R packages. Differentially expressed genes (DEGs) of HICGs were identified using the criteria of |log2FC| > 0.5 and an adjusted *p*-value (padj) < 0.05. Volcano plots were generated using the ggplot2 package to visualize DEGs.

### Weighted gene co-expression network analysis

To identify gene modules associated with LF, weighted gene co-expression network analysis (WGCNA) was performed using the WGCNA package (v1.73) on the GSE49541 dataset (Supplementary Table 1). An adjacency matrix was constructed and converted into a topological overlap matrix, followed by hierarchical clustering to define modules. The module eigengenes were then correlated with clinical traits (fibrotic vs. control), and significant modules were identified based on these correlations.

### Validation and diagnostic evaluation of HICGs

The intersection of significant modules identified from WGCNA, HICGs, and DEGs from three transcriptomic datasets was determined using the VennDiagram package. Expression profiles of the selected genes were visualized using violin plots, generated with the ggpubr package. To evaluate their diagnostic potential, receiver operating characteristic (ROC) curves were constructed using the pROC package, and the area under the curve (AUC) for each gene was calculated with 95% confidence intervals.

### Single-cell transcriptomics analysis

Single-cell RNA sequencing data for LF were retrieved from the Single Cell Portal (https://singlecell.broadinstitute.org/single_cell), specifically from patients with alcoholic cirrhosis (SCP2154), which included 328,783 cells.

### Co-expression analysis of HICGs in LF

Expression patterns were visualized through hierarchical clustering heatmaps, generated with the pheatmap package. To explore co-expression relationships, Pearson correlation coefficients were calculated, and the resulting correlation matrix was visualized as heatmaps using the corrplot package (v0.95).

### Functional enrichment analysis of HICGs

Functional enrichment of the identified HICGs was performed using the clusterProfiler package (v4.12.6) in R, including Gene Ontology (GO), Kyoto Encyclopedia of Genes and Genomes (KEGG), and Gene Set Enrichment Analysis (GSEA). Significant GO terms and pathways were identified with a *p*-adjusted value (*p*-adj) < 0.05, applying the Benjamini-Hochberg correction. For GSEA, genes were ranked according to their correlation with the expression of HICG, and pathways with normalized enrichment scores > 1 and false discovery rates < 0.25 were considered significant.

### Immune profiling and correlation with HICGs

Immune cell composition in fibrotic and non-fibrotic liver tissues was estimated using the CIBERSORT algorithm (v1.03) with the LM22 gene signature. Differences in immune cell proportions were statistically compared, and correlations between hub HICGs and immune subsets were evaluated using Pearson correlation analysis.

### Molecular docking

Candidate compounds were obtained from the DSigDB database [17] and filtered to prioritize FDA-approved or clinically validated drugs with acceptable safety. Other exclusions comprised eight chemotherapeutic agents (vincristine, cytarabine, vorinostat, tamoxifen, retinoic acid, irinotecan, gemcitabine, and decitabine), along with sex hormones, corticosteroids, anti-infectives, and diuretics, based on their distinct mechanisms or potential adverse effects. Protein structures were retrieved from UniProt (https://www.uniprot.org, accessed November 4, 2025) and ligand conformations were obtained from PubChem (https://pubchem.ncbi.nlm.nih.gov, accessed November 4, 2025). Docking simulations were performed using the CB-DOCK2 docking server (version 2 of CB-DOCK, accessed November 4, 2025) [18, 19] under blind docking conditions, and binding affinity was assessed based on Vina scores.

### Immunohistochemistry

Human liver tissue samples were obtained from the Fourth Hospital of Changsha, with control tissues sourced from non-cirrhotic cases, including hepatic trauma resections and cholecystectomy-derived liver sections. Liver tissue samples from bile duct ligation-induced fibrotic mice were kindly provided by Dr. Yalan Deng (Xiangya Hospital) [20] and validated by Hematoxylin and eosin (H&E) and Masson’s trichrome staining. Immunohistochemical staining was performed using standard protocols [21], with antigen retrieval in Tris-EDTA buffer (pH 9.0). The primary antibody against GJA1 (Servicebio, GB2234-50) was diluted 1:300. The expression levels of GJA1 were assessed based on staining intensity and abundance, with images captured at 200× magnification.

## Results

### Data integration and identification of HICGs in LF

**Fig. 1:**
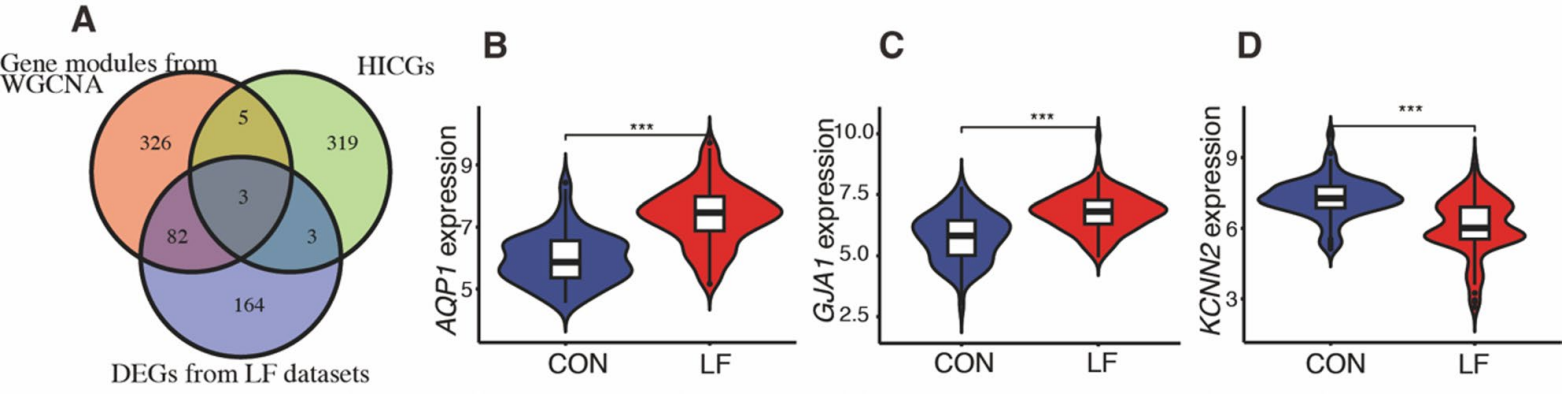
Intersection analysis and validation of hub HICGs in LF. (**A**) Venn diagram showing the overlap among fibrosis-associated genes identified by WGCNA, differentially expressed genes, and HICGs, identifying three shared hub genes: *AQP1*, *GJA1*, and *KCNN2*. (**B**–**D**) Violin plots illustrating the expression levels of *AQP1* (**B**), *GJA1* (**C**), and *KCNN2* (**D**) in non-fibrotic and fibrotic liver tissues (all *p* < 0.001)

Transcriptomic datasets from the GEO (GSE14323, GSE84044, and GSE197112), containing liver tissue samples from both fibrotic and non-fibrotic individuals, were analyzed. Principal component analysis initially revealed significant batch effects (Supplementary Fig. 1A), which were effectively corrected after normalization to ensure data comparability (Supplementary Fig. 1B). Differential expression analysis identified HICGs associated with LF (Supplementary Fig. 1C). Furthermore, WGCNA on the GSE49541 dataset identified three key modules, among which the blue and gray modules displayed significant differential expression (Supplementary Fig. 2). This integrated analysis identified three hub HICGs: aquaporin-1 (*AQP1*), gap junction protein alpha 1 (*GJA1*), and potassium calcium-activated channel subfamily N member 2 *(KCNN2*) (Fig. 1A). As shown in the violin plots (Fig. 1B–D), *KCNN2* was significantly downregulated in fibrotic liver tissues, whereas *AQP1* and *GJA1* were upregulated. Importantly, subgroup analysis across different LF etiologies (hepatitis B, hepatitis C, and NAFLD) demonstrated that these expression trends were consistent (Supplementary Fig. 3).

**Fig. 2:**
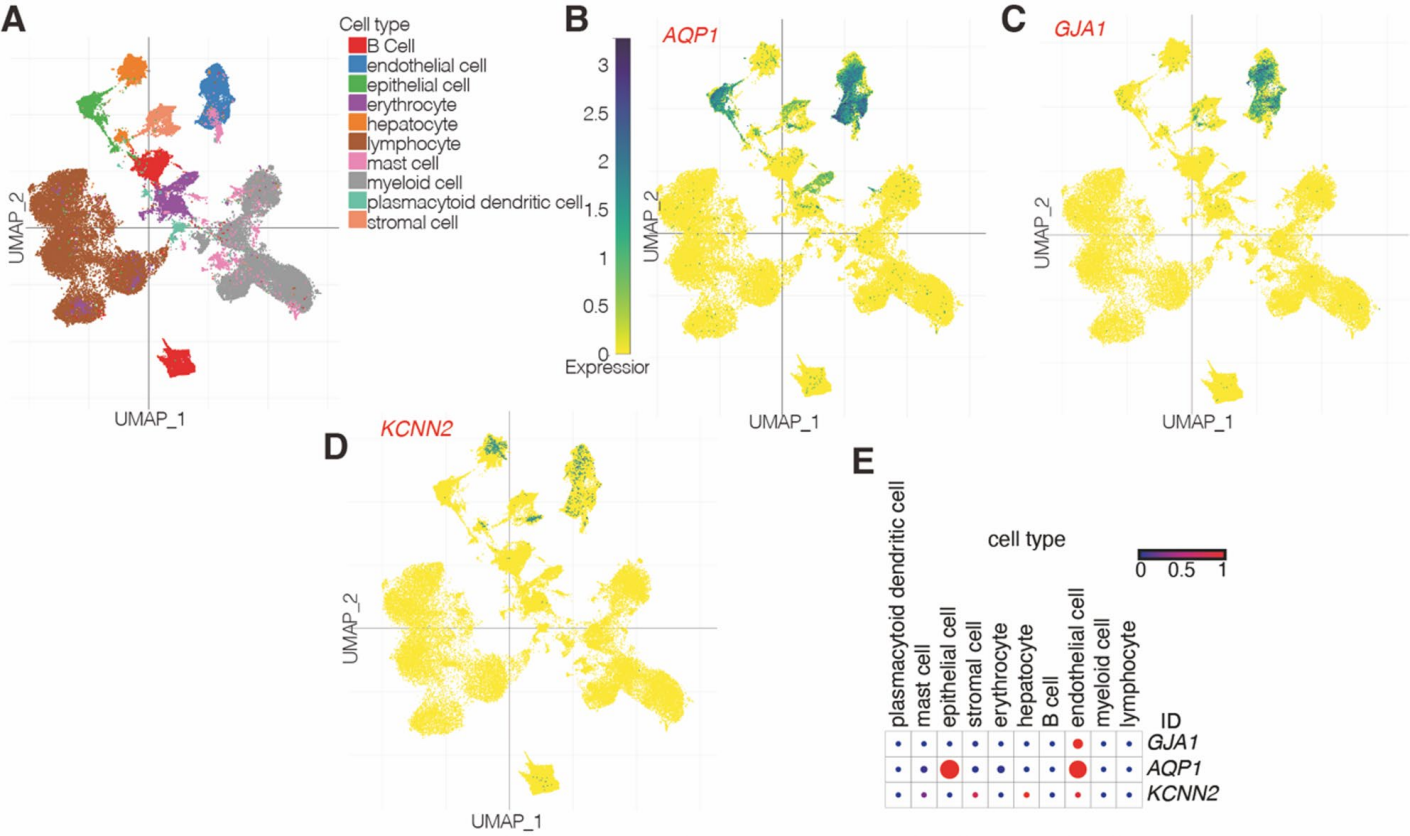
Single-cell transcriptomic profiling of three hub HICGs in alcoholic cirrhosis. (**A**) UMAP plot illustrating the classification of major hepatic cell populations in single-cell RNA sequencing data from human alcoholic cirrhosis. (**B**–**D**) UMAP visualization of the expression patterns of *AQP1*, *GJA1*, and *KCNN2*. (**E**) Dot plot showing the cell-type distribution and relative expression levels of *AQP1*, *GJA1*, and *KCNN2*

Single-cell RNA sequencing analysis of alcoholic LC revealed that *AQP1* is primarily expressed in epithelial cells, endothelial cells, erythrocytes, and stromal cells (Fig. 2A and B), while *GJA1* was predominantly found in endothelial cells (Fig. 2A and C). *KCNN2* was enriched in epithelial cells (Fig. 2A and D). The expression patterns of these genes in liver tissues are presented in Fig. 2E. These results demonstrate distinct expression patterns of *AQP1*, *GJA1*, and *KCNN2* in LF tissues.

### Clinical application of HICGs in LF

**Fig. 3:**
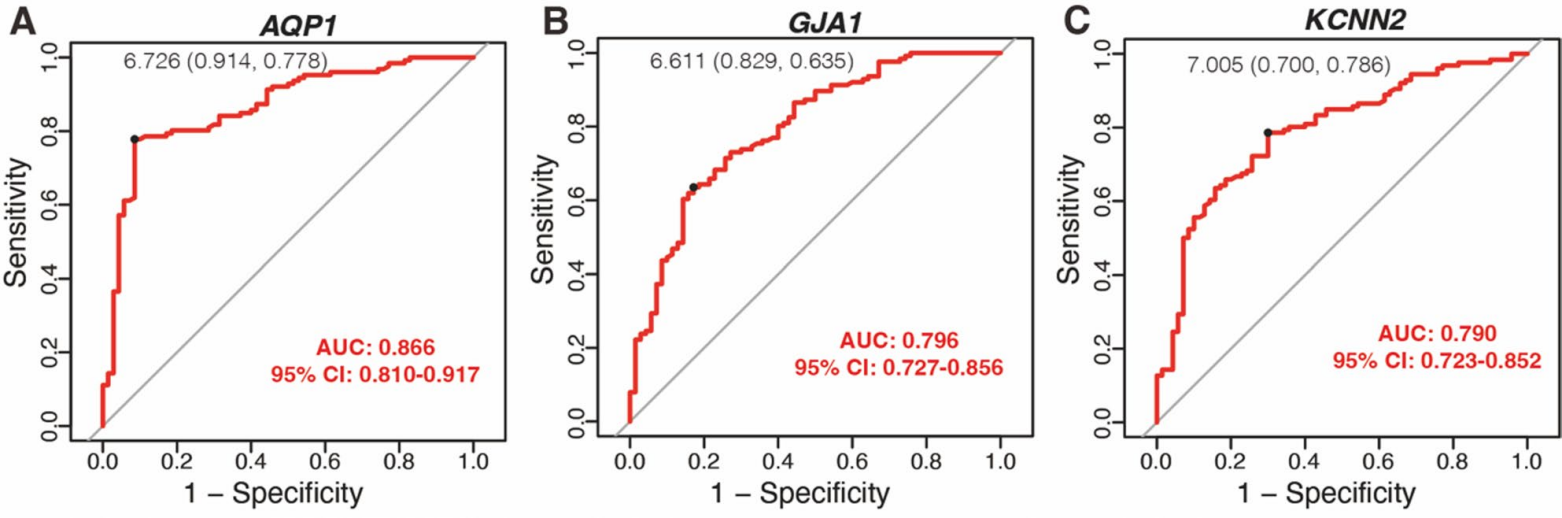
Diagnostic value of HICGs in LF. ROC curves evaluating the diagnostic accuracy of three HICGs: *AQP1* (**A**), *GJA1* (**B**), and *KCNN2* (**C**) in distinguishing LF from non-fibrotic samples

**Fig. 4:**
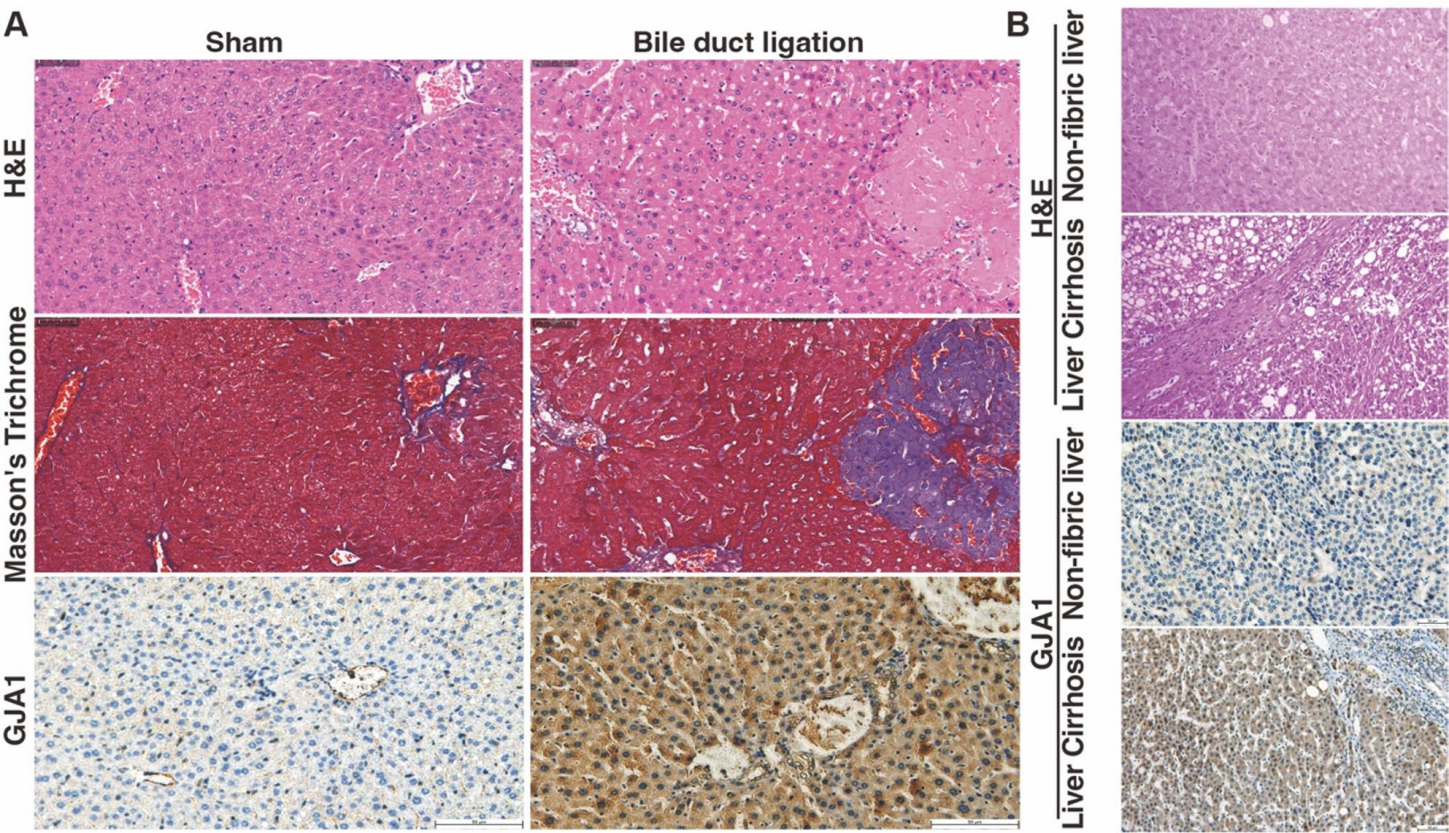
Validation the expression of GJA1 in mouse and human liver tissues. (**A**) Representative H&E, Masson’s trichrome, and GJA1 immunohistochemistry images from bile duct ligation-induced fibrosis mouse and control mouse livers (200×). (**B**) H&E staining and GJA1 immunohistochemistry in normal and cirrhotic human liver tissues (200×)

To assess the clinical diagnostic value of the identified HICGs for LF, ROC curve analysis was performed. The AUC values for *AQP1*, *GJA1*, and *KCNN2* were 0.866, 0.796, and 0.790, respectively (Fig. 3A-C), indicating their strong diagnostic potential for LF. To further validate the expression of these HICGs in other LF models, immunohistochemistry for GJA1 was conducted. H&E and Masson’s trichrome staining confirmed successful induction of LF in bile duct ligation-treated mice (Fig. 4A). Immunohistochemistry showed strong positive staining for GJA1 in fibrotic liver tissues, particularly in hepatocytes and biliary epithelial cells in the portal area, compared to weak staining in the control group (Fig. 4A). In paired non-cirrhotic and cirrhotic liver tissue samples, immunohistochemistry showed lower expression of GJA1 in non-cirrhotic tissues compared to cirrhotic liver, particularly in hepatocytes (Fig. 4B).

To further evaluate the generalizability of these biomarkers across different etiologies of LF, subgroup ROC analyses were conducted (Supplementary Table 2). *AQP1* consistently demonstrated excellent diagnostic performance (all AUCs ≥ 0.949), with high specificity (≥ 0.906) across all subgroups. In addition, subgroup analysis indicated that *GJA1* and *KCNN2* exhibited the highest diagnostic value for predicting chronic hepatitis C virus-associated LF, while they also showed favorable efficacy in LF induced by hepatitis B virus infection and NAFLD (all AUCs ≥ 0.800). These findings suggest that *AQP1*, *GJA1*, and *KCNN2* may serve as valuable clinical biomarkers for LF.

### Co-expression patterns of HICGs

**Fig. 5:**
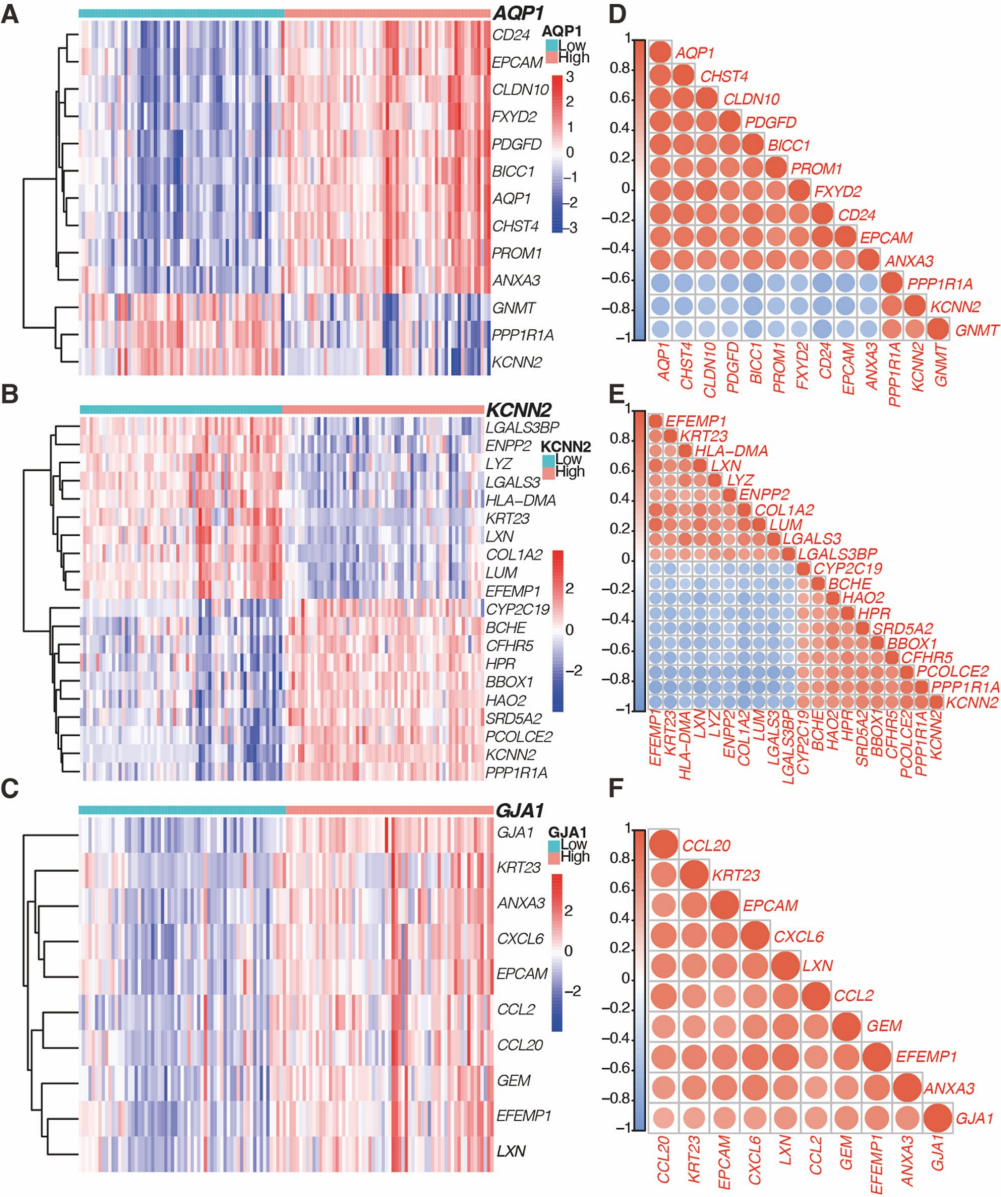
Co-expression patterns and correlation analysis of HICGs and related genes. (**A**–**C**) Heatmaps display the top correlated genes associated with the expression of *AQP1*, *KCNN2*, and *GJA1*, respectively. (**D**–**F**) Correlation matrices show the Spearman correlation coefficients among each HICG and its co-expressed genes

To explore the co-expression patterns of these hub HICGs, a heatmap was generated to illustrate the correlations with related genes (Fig. 5A). Negative co-expression was observed between *AQP1* and *KCNN2*, *PPP1RA*, and *GNMT*, while positive co-expression was found between *KCNN2* and *PPP1RA* (Fig. 5B). For *GJA1*, significant positive co-expression was observed with chemokines *CCL2* and *CCL20*, as well as cell matrix proteins *EPCAM* and *EFEMP1* (Fig. 5C). Spearman correlation analysis (Fig. 5D–F) revealed that these genes exhibited the highest co-expression with HICGs, suggesting that they may collectively contribute to LF progression.

### Enrichment of HICGs in biological processes and pathways

To elucidate the biological roles of the identified HICGs, GO and KEGG enrichment analyses were conducted. GO analysis revealed that *AQP1* is primarily involved in ECM organization, external structure organization, and collagen-containing ECM (Supplementary Fig. 4A). *GJA1* is associated with chemokine-mediated signaling, tight junctions, and cytokine receptor binding (Supplementary Fig. 4B), while *KCNN2* is involved in chemokine signaling and collagen-containing ECM (Supplementary Fig. 4C). KEGG pathway analysis highlighted that *AQP1* is implicated in focal adhesion, bile secretion, and ECM-receptor interactions (Supplementary Fig. 4D). *GJA1* participates in IL-17 signaling, chemokine signaling, TNF signaling, and cytokine-receptor interactions (Supplementary Fig. 4E), while *KCNN2* is primarily involved in cytokine-cytokine receptor interactions and chemokine signaling (Supplementary Fig. 4F).

**Fig. 6:**
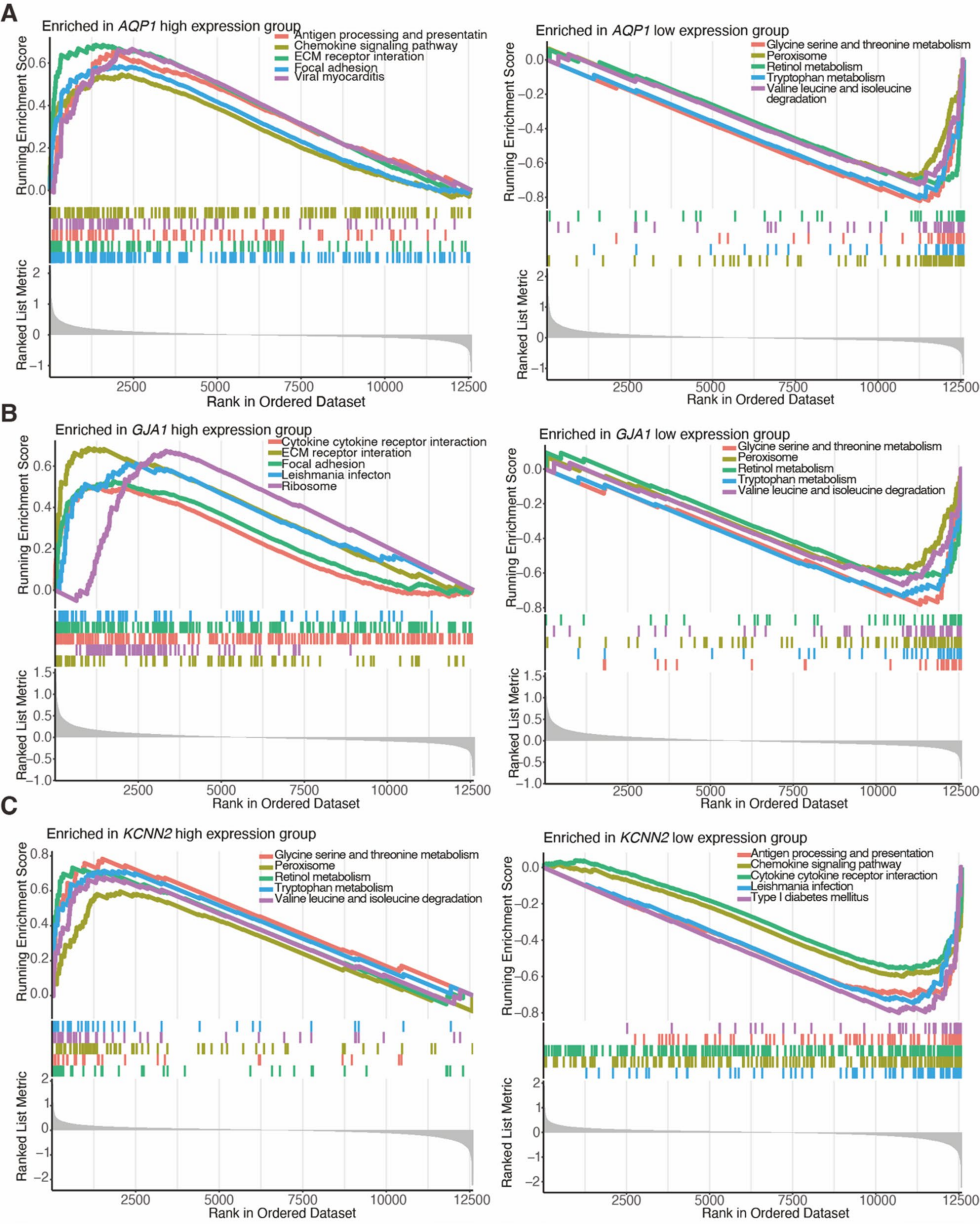
GSEA of HICGs in LF. GSEA plots illustrating representative signaling pathways enriched in high- and low-expression groups based on the expression of (**A**) *AQP1*, (**B**) *GJA1*, and (**C**) *KCNN2*

GSEA further corroborated these findings, showing that high expression of *AQP1* and low expression of *KCNN2* were associated with antigen processing, cytokine signaling, receptor interactions, and focal adhesion (Fig. 6A and C). *GJA1*’s GSEA results were consistent with KEGG, showing its involvement in cytokine-cytokine receptor interactions and ECM-receptor interactions (Fig. 6B). Low expression of *AQP1* and *GJA1*, along with high expression of *KCNN2*, was primarily enriched in various cellular metabolic processes, such as lysine, serine, and threonine metabolism, leucine and isoleucine degradation, and tryptophan metabolism (Fig. 6A and C). These enrichment analyses collectively revealed significant associations between HICGs and pathways related to immune signaling and metabolic reprogramming in LF.

### PPIs and reactome pathway of HICGs

To further explore the biological roles of HICGs from a proteomics perspective, the PPI networks for AQP1, GJA1, and KCNN2 were analyzed using the STRING database. Cluster and enrichment analyses showed that AQP1 interacts mainly with proteins involved in passive transport by aquaporins (Supplementary Fig. 5A and Supplementary Fig. 6A). KCNN2 interacts with components of Ca²⁺-activated K⁺ channels (Supplementary Fig. 5B and Supplementary Fig. 6B), and GJA1 interacts with constituents mediating gap junction trafficking, regulation, and assembly (Supplementary Fig. 5C and Supplementary Fig. 6C).

### Immune microenvironment landscape of HICGs

**Fig. 7:**
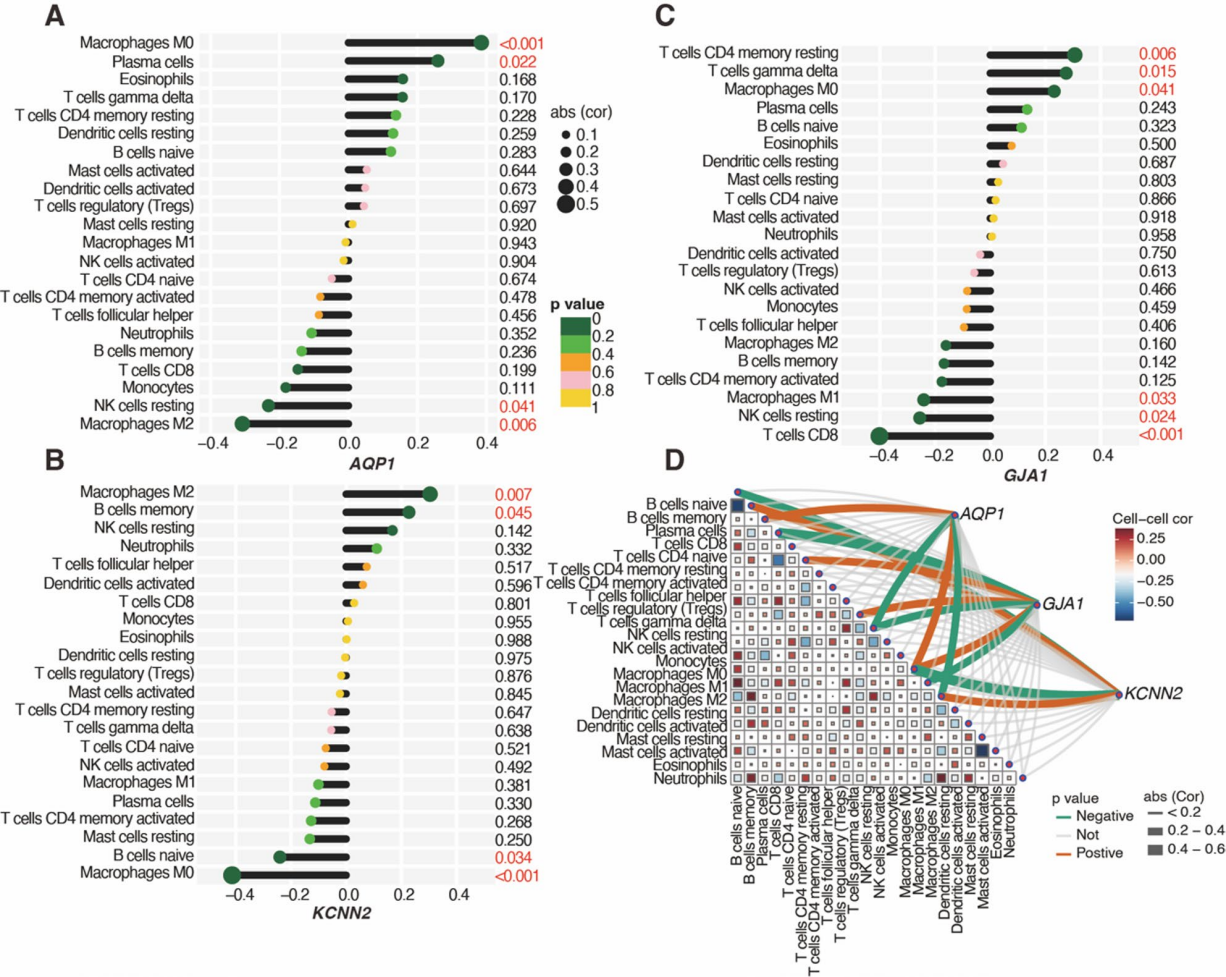
Correlation analysis between hub HICGs and immune cell infiltration in LF. Spearman correlation analysis between the expression levels of (**A**) *AQP1*, (**B**) *KCNN2* and (**C**) *GJA1* and immune cell infiltration patterns in LF tissues. (**D**) Immune correlation network showing interactions between three HICGs and immune cells

Given the observed associations between HICGs and immune pathways in GO and GSEA analyses, the immune features of LF were further analyzed. The estimated proportions of 22 immune cell types in fibrotic and non-fibrotic liver samples are shown in Supplementary Fig. 7. Correlation analysis unveiled significant associations between the expression levels of HICGs and immune cell abundance (Fig. 7A and C). Notably, the expression of *AQP1* was positively correlated with M0 macrophages and plasma cells but negatively correlated with resting NK cells and M2 macrophages. In contrast, the expression of *KCNN2* was positively correlated with M2 macrophages and memory B cells but negatively correlated with naïve B cells and M0 macrophages. The expression of *GJA1* correlated positively with resting CD4 memory T cells, gamma-delta T cells, and M0 macrophage infiltration, but negatively with M1 macrophages, resting NK cells, and CD8 T cells. These results demonstrate significant associations between the expression of HICGs and immune cell infiltration, particularly M2 and M0 macrophages and resting NK cells.

### Molecular docking simulation for drug targeting HICGs

**Fig. 8:**
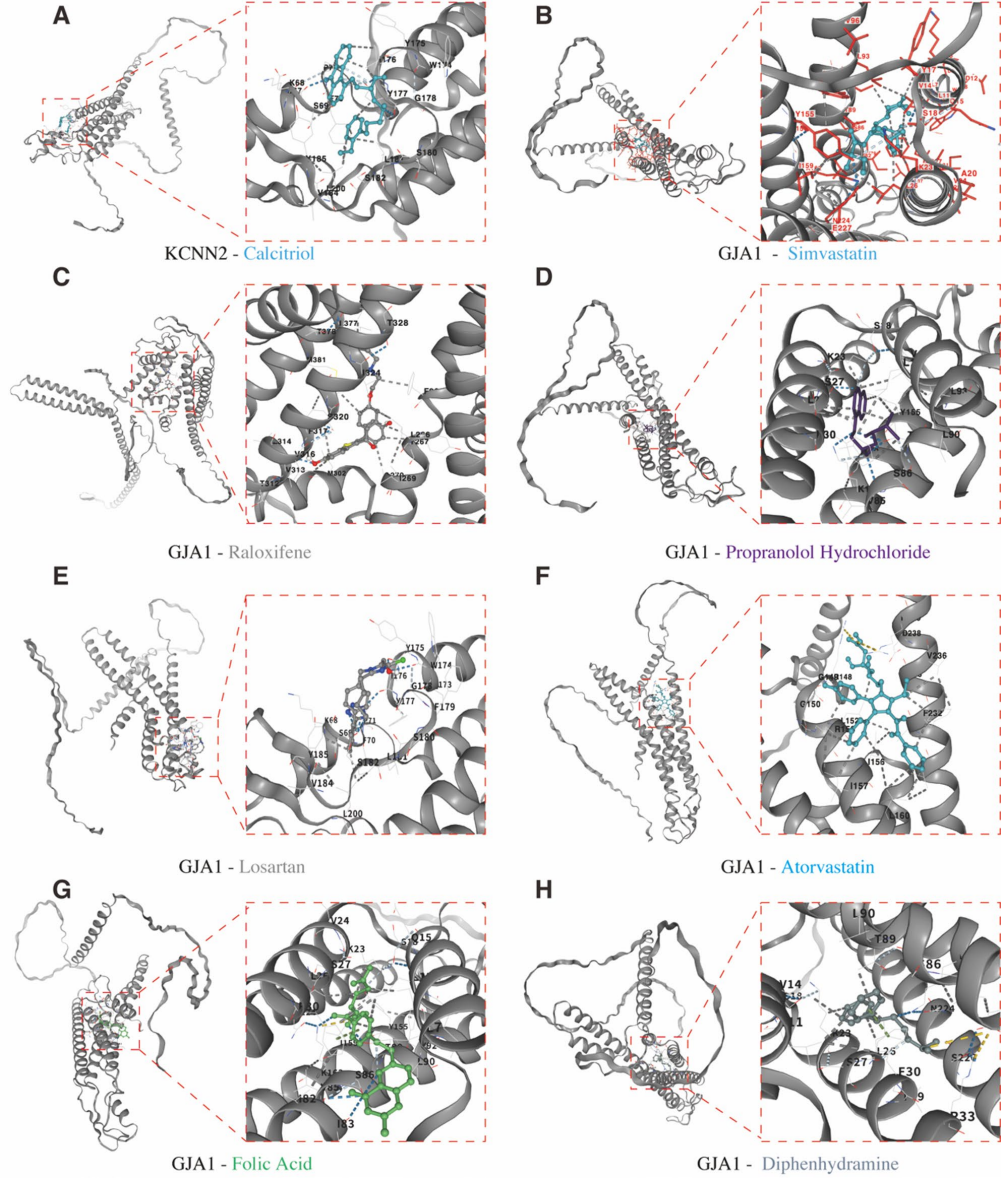
Molecular docking of potential drugs with GJA1 and KCNN2. (**A**) Predicted interaction between Calcitriol and KCNN2. Docking models illustrating the binding of Simvastatin (**B**), Raloxifene (**C**), Propranolol hydrochloride (**D**), Losartan (**E**), Atorvastatin (**F**), Folic acid (**G**), Diphenhydramine (**H**) to GJA1. Each panel shows the overall protein conformation and the magnified view of the binding pocket

To explore the therapeutic potential of targeting HICGs in LF, molecular docking simulations were conducted. After screening, 19 clinically relevant drugs were identified as potential candidates targeting GJA1 and KCNN2. Vina scoring revealed 16 drugs with favorable binding affinities: Calcitriol, Simvastatin, Rosiglitazone, Raloxifene, Propranolol Hydrochloride, Valsartan, Losartan, Cerivastatin, Carvedilol, Calcium DL-Pantothenate, Atorvastatin, Ganciclovir, Folic Acid, Diphenhydramine, and Diazepam (Supplementary Table 3). These drugs mainly included antihypertensive agents, such as non-selective β-adrenergic receptor blocker (NSBB) and angiotensin II receptor blocker (ARB), as well as lipid-lowering statins. The simulation structures of drug-protein interactions with Vina scores ≤ -7 are shown in Fig. 8. These findings identify multiple clinically relevant compounds with favorable binding affinity toward HICGs.

## Discussion

LF is a common pathological outcome of various chronic liver diseases, characterized by excessive deposition and abnormal remodeling of ECM components. This leads to hepatic structural distortion and impaired liver function. Ion channels, which regulate the transport of ions and mediate signal transduction in response to extracellular stimuli, are essential for maintaining liver homeostasis and influencing disease progression [22]. In this study, we comprehensively analyzed the expression profiles of HICGs in LF and explored their potential roles in fibrosis progression. Our results indicate that *AQP1* and *GJA1* were significantly upregulated, while *KCNN2* was downregulated in fibrotic tissues compared to normal liver. These alterations in ion channels likely influence intercellular signaling, receptor interactions, ECM remodeling, cellular metabolism, antigen presentation, and immune cell infiltration, particularly M0 and M2 macrophages, all of which contribute to the fibrotic process. Furthermore, based on molecular docking analyses targeting KCNN2 and GJA1, we identified 16 potential drugs that may impact LF. These findings not only identify three biomarkers involved in LF progression but also provide new insights into the role of ion channel expression in fibrosis, offering potential targets for the development of antifibrotic therapies.

AQP1 is predominantly localized in the arterial capillaries surrounding the portal vein and intrahepatic bile ducts under normal physiological conditions, where it plays a key role in bile secretion in the liver, bile duct, and gallbladder levels [23]. Adenoviral-mediated overexpression of *AQP1* has been shown to normalize bile flow and bile acid excretion in cholestatic liver diseases, suggesting *AQP1* as a potential therapeutic target for cholestasis [24–26]. Our results show that *AQP1* is upregulated in LC, with its high expression levels correlating with an increased ability to predict LF. In cirrhosis, AQP1 is induced and localized in sinusoidal endothelial cells, proliferating bile ducts, and some arterial capillaries connected to sinusoids, where it contributes to the proliferation of hepatic microcapillaries and portal hypertension [14, 27]. AQP1 is also significantly upregulated in microvascular membranes in hepatocellular carcinoma, correlating with tumor progression and prognosis [28]. Due to the abundant expression of AQP1 in other organs such as the kidneys [29], conventional drugs targeting AQP1 may lead to adverse effects, including osmotic imbalance, electrolyte disturbances, and increased intracranial pressure. Therefore, liver-specific targeting strategies for AQP1 in hepatic endothelial cells may provide a novel approach for reversing LF and portal hypertension.

In addition, our bioinformatics analysis of GEO datasets and subsequent immunohistochemical validation revealed a significant upregulation of GJA1, a protein responsible for high-speed signal transmission in liver cells. KEGG and GSEA pathway analyses suggest that *GJA1* plays a central role in regulating cytokine-receptor interactions and ECM-receptor signaling, key pathways involved in fibrosis progression. Under physiological conditions, *GJA1* is largely restricted to non-parenchymal liver cells [30], while gap junctions between hepatocytes primarily consist of CX32 and CX26, mediating the exchange of small molecules between cytosols. In models of LC induced by bile duct ligation, the expression of CX32 and CX26 is downregulated, while GJA1 is upregulated in hepatocytes and non-parenchymal cells (including endothelial cells), consistent with our findings. This upregulation is further exacerbated following lipopolysaccharide stimulation [31]. Studies have shown that the expression of GJA1 is positively correlated with elevated tumor necrosis factor-alpha levels and the activation of the NF-κB pathway [32–34]. Intervention with infliximab significantly reduced the expression of GJA1 protein in bile duct ligation rat models, while treatment with anti-GJA1 peptides led to widespread hepatocyte necrosis and portal-portal bridging fibrosis [31]. Notably, *GJA1* deficiency exacerbates liver injury and negatively impacts prognosis [35]. Additionally, studies in a CCl₄-induced chronic liver injury model have shown that *GJA1* knockout mice exhibited more severe LF, less severe necrotic inflammation, and lower serum aminotransferase levels [36]. Although these studies suggest that upregulation of *GJA1* may be an adaptive protective response to enhance cell communication during LF [31], the exact role of *GJA1*, particularly in hepatocytes, remains to be clarified.

Our study also identified *KCNN2* as a key downregulated ion channel in LF. While the role of *KCNN2* has been documented in cardiac fibrosis and atrial fibrillation [37], its specific function and underlying mechanisms in LF are not well understood. KCNN2 is expressed in the serous membranes of cholangiocytes and plays a critical role in bile secretion, regulated by calcium ion stimulation [38]. The downregulation of *KCNN2* in LF may disrupt ion homeostasis in bile flow, potentially exacerbating cholestasis, a major contributor to LF progression. In the process of myocardial fibrosis remodeling, *KCNN2* has been involved in the TGF-β signaling pathway [39], which is a key cytokine in hepatic stellate cell activation. Given that KEGG analysis revealed that *KCNN2* is involved in cytokine-cytokine receptor interactions and chemokine signaling pathways, we hypothesize that *KCNN2* may similarly play a role in LF through analogous signaling mechanisms. Further studies using various LF models and electrophysiology techniques will be necessary to elucidate the specific functions and mechanisms of *KCNN2* in LF.

In addition to identifying HICGs in LF, molecular docking simulations suggest that several drugs, including statins and antihypertensive agents (e.g., propranolol and valsartan), may target KCNN2 and GJA1. Currently, these drugs are common treatments for portal hypertension although statins are associated with potential liver dysfunction risks. Recent large-scale cohort studies have shown that statin use in chronic liver disease patients reduces the risk of liver decompensation and hepatocellular carcinoma by 22% and 33%, respectively. Moreover, statins may slow the progression of LF [40]. In a dose- and age-dependent manner, statin use is associated with improved clinical outcomes in LC [41]. Additionally, a cross-sectional survey of patients with metabolic-associated fatty liver disease showed that the use of antidiabetic and lipid-lowering medications is linked to reduced serum LF biomarkers such as aspartate aminotransferase-to-platelet ratio index and fibrosis-4 index [42]. Mechanistically, statins not only lower cholesterol but also exert antifibrotic effects by reducing portal pressure, improving endothelial dysfunction, and enhancing immune cell function. Simvastatin treatment selectively increases NO availability in cirrhotic liver circulation, improving vascular disturbances contributing to portal hypertension [43]. In vivo experiments have demonstrated that pitavastatin enhances *Nrf2* and *Ho1*, inhibits inflammation (e.g., *Mda*, *Nfκb1*), and effectively reduces LF by suppressing the expression of p-AKT [44]. Statins have also been shown to restore hepatic NK cell metabolic activity [45] and enhance regulatory T cell function [46]. Hence, we hypothesize that statins and NSBBs may act on GJA1 to regulate intercellular communication and mediate their anti-cirrhotic effects, providing a novel mechanism to explain antifibrotic drug action.

Despite the comprehensive multi-omics bioinformatic analyses performed in this study, several limitations should be acknowledged. First, although normalization and batch effect correction were applied to integrate three datasets, residual heterogeneity cannot be fully eliminated, and future studies based on larger, single-batch cohorts are required to further validate. Second, to facilitate intersection of DEGs from the predefined HICG set with fibrosis-related modules identified by WGCNA, relatively loose thresholds of |log₂FC| > 0.5 and *p* < 0.05 were applied; while widely accepted in transcriptomic studies [47, 48] and allows the detection of functionally relevant genes with mild-to-moderate expression changes, this approach may increase false positives and therefore warrants validation in independent datasets. In addition, using bulk transcriptomic data may obscure cell-type–specific expression changes of HICGs, particularly in non-parenchymal hepatic cells, suggesting the need for future studies incorporating spatial or single-cell transcriptomics. From a biological and clinical perspective, subgroup analyses support the promising diagnostic value of HICGs across common LF etiologies; nevertheless, nevertheless, the limited sample size within each subgroup risks statistical bias, and the pathogenesis of LF may differ across etiologies, so validation in larger, etiology-specific cohorts—including other common causes—is warranted. Moreover, the precise mechanisms by which changes in ion channel expression regulate LF through the immune microenvironment remain unclear and require further in vitro and in vivo investigation. Finally, although molecular docking simulations identified potential drugs targeting HICGs, their ability to modulate ion channel activity and exert antifibrotic effects awaits experimental and clinical validation.

## Conclusion

This study identifies three hub HICGs—*AQP1*, *GJA1*, and *KCNN2*—that are involved in the progression of LF. *AQP1* and *GJA1* are upregulated, while *KCNN2* is downregulated in fibrotic tissues. These ion channels regulate fibrosis through mechanisms such as bile secretion, ECM-receptor signaling, and immune cell infiltration. We identified 16 potential drugs, including statins, NSBBs, and ARBs, as promising candidates for targeting these HICGs and mitigating LF. These findings provide a foundation for further research into the role of ion channels in LF and offer new perspectives on therapeutic strategies for LF.

## Supplementary Information

Below is the link to the electronic supplementary material.

Supplementary Material 1

## Data Availability

The datasets analyzed in this study are publicly available data and annotated with the source. The data and material used during the current study are available from the corresponding author upon reasonable request.

## Abbreviations

AQP: Aquaporins
ARB: Angiotensin II receptor blocker
AUC: Area under the curve
DEGs: Differentially expressed genes
ECM: Extracellular matrix
GEO: Gene Expression Omnibus
GO: Gene Ontology
GSEA: Gene Set Enrichment Analysis
H&E: Hematoxylin-eosin
LC: Liver cirrhosis
LF: Liver fibrosis
KEGG: Kyoto Encyclopedia of Genes and Genomes
NAFLD: Non-alcoholic fatty liver disease
NSBB: Non-selective β-adrenergic receptor blocker
PPI: Protein–protein Interaction
ROC: Receiver operating characteristic
WGCNA: Weighted gene co-expression network analysis
