# Supplementary material for: Ion channel gene signature for diagnosis and antifibrotic therapy in liver fibrosis
Source: J Transl Med. 2026 Feb 16;24:295. doi: 10.1186/s12967-026-07856-1 (PMC12930822; doi:10.1186/s12967-026-07856-1)
Supplement: Supplementary file 1 — Supplementary Material 1 [file 12967_2026_7856_MOESM1_ESM.docx]

**Ion Channel Gene Signature for Diagnosis and Antifibrotic Therapy in Liver Fibrosis**

**Supplementary Figures:**


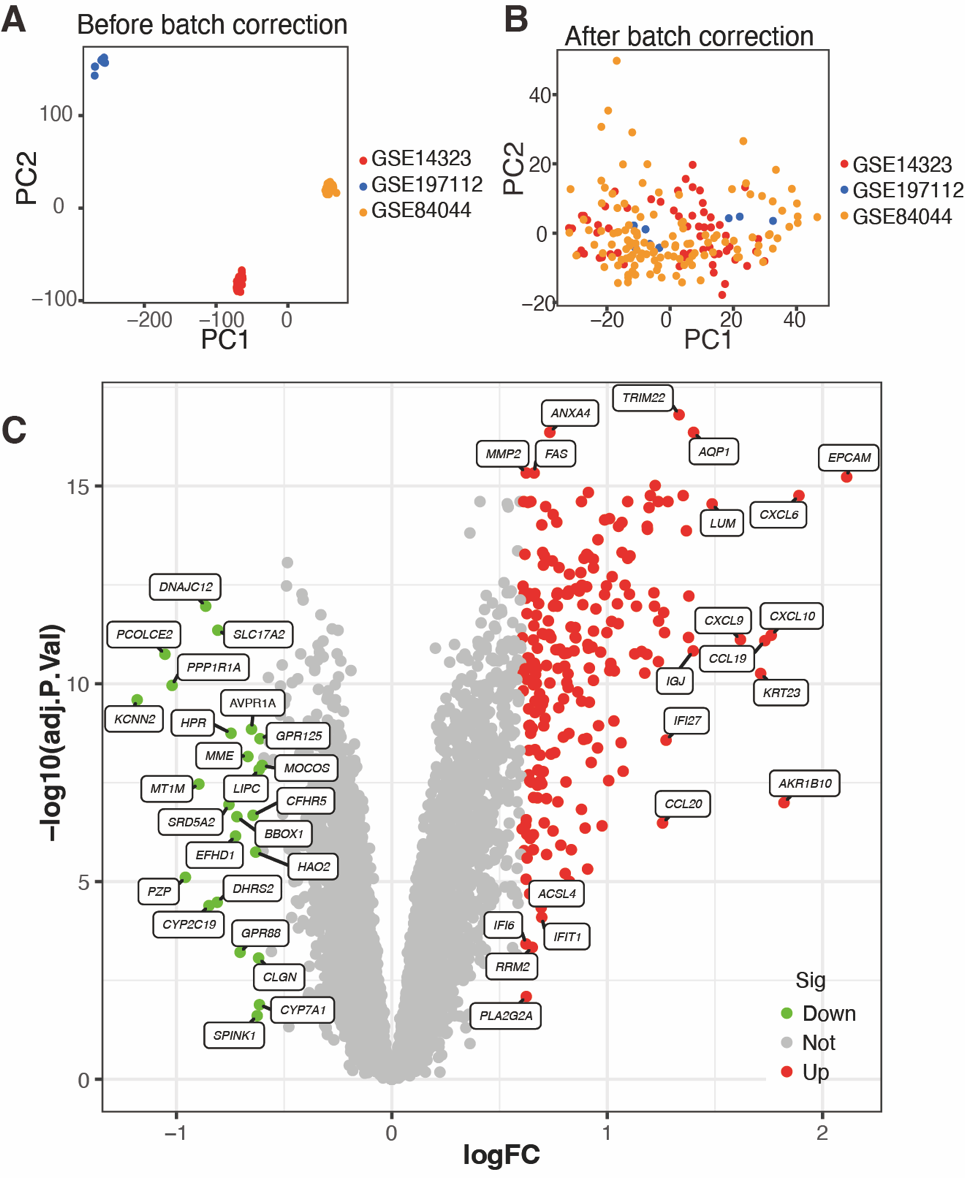


Supplementary Figure 1. Batch correction and DEGs of HICGs in LF.

(A) Principal component analysis plots illustrating separation across three GEO cohorts before batch correction. (B) Principal component analysis plots showing improved dataset integration after batch correction. (C) Volcano plot of DEGs of HICGs between LF and non-fibrosis groups.


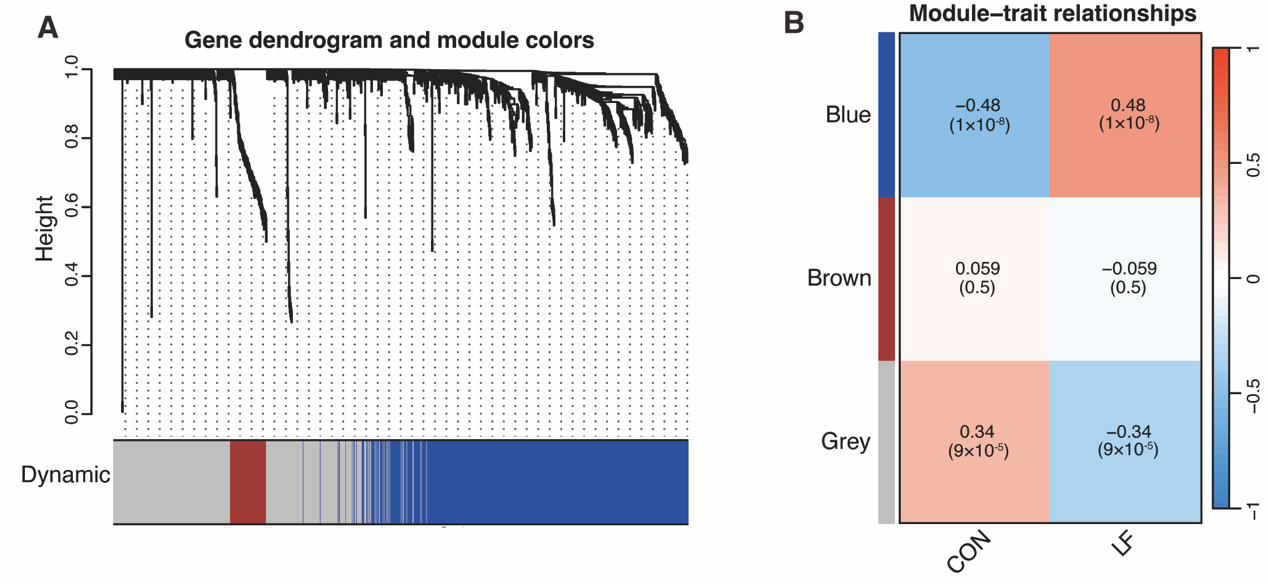


Supplementary Figure 2. WGCNA analysis identifies key modules associated with LF and HICGs expression in the GSE49541 dataset.

(A) Gene dendrogram obtained by hierarchical clustering. (B) Heatmap of module–trait correlations.


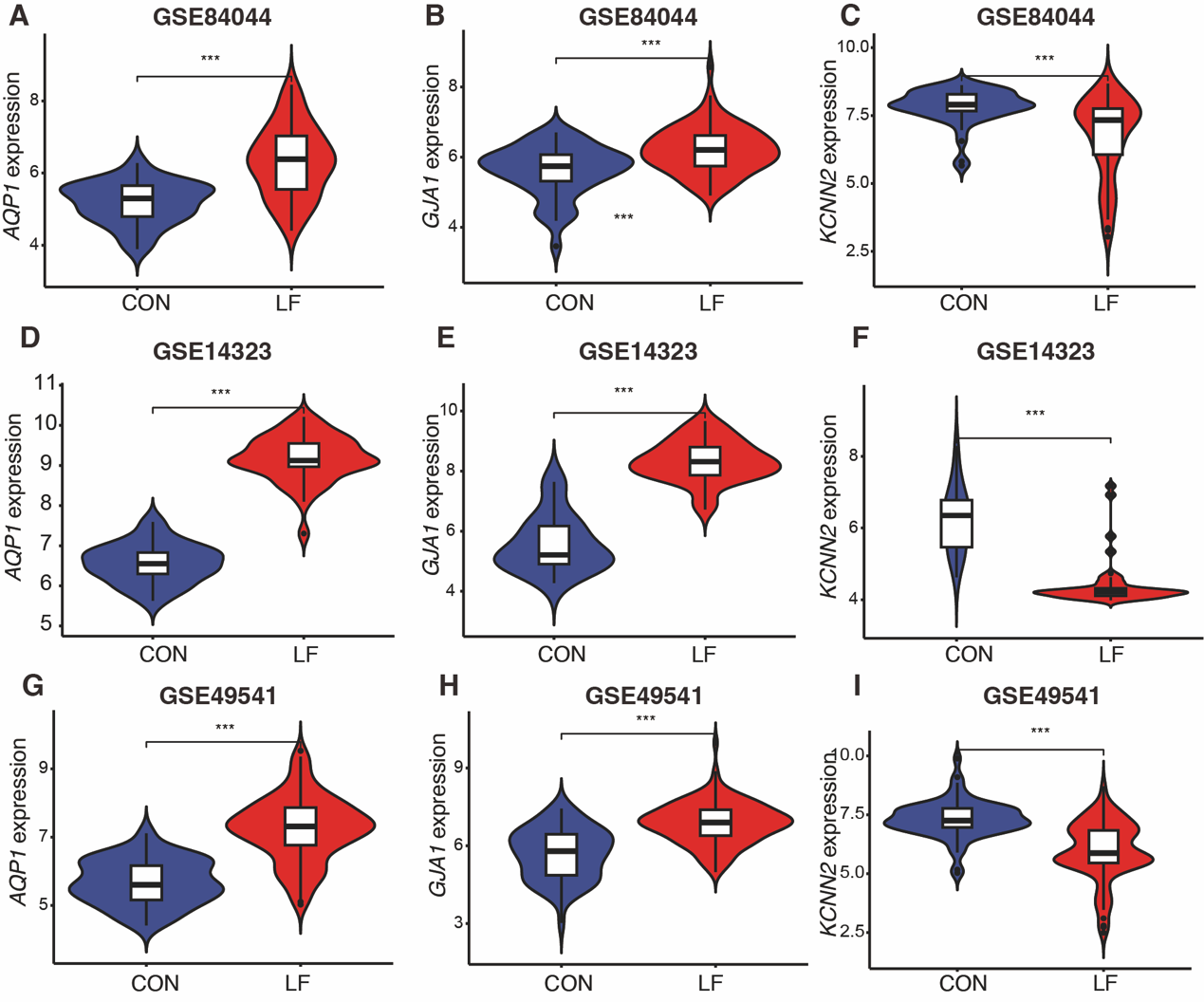


Supplementary Figure 3. Expression patterns of hub HICGs across LF datasets with different etiologies.

Violin plots illustrate the expression distributions of *AQP1* (A, D, and G), *GJA1* (B, E, and H), and *KCNN2* (C, F, and I) in three independent LF datasets GSE84044 (A-C), GSE14323 (D-F), and GSE49541 (G-I).


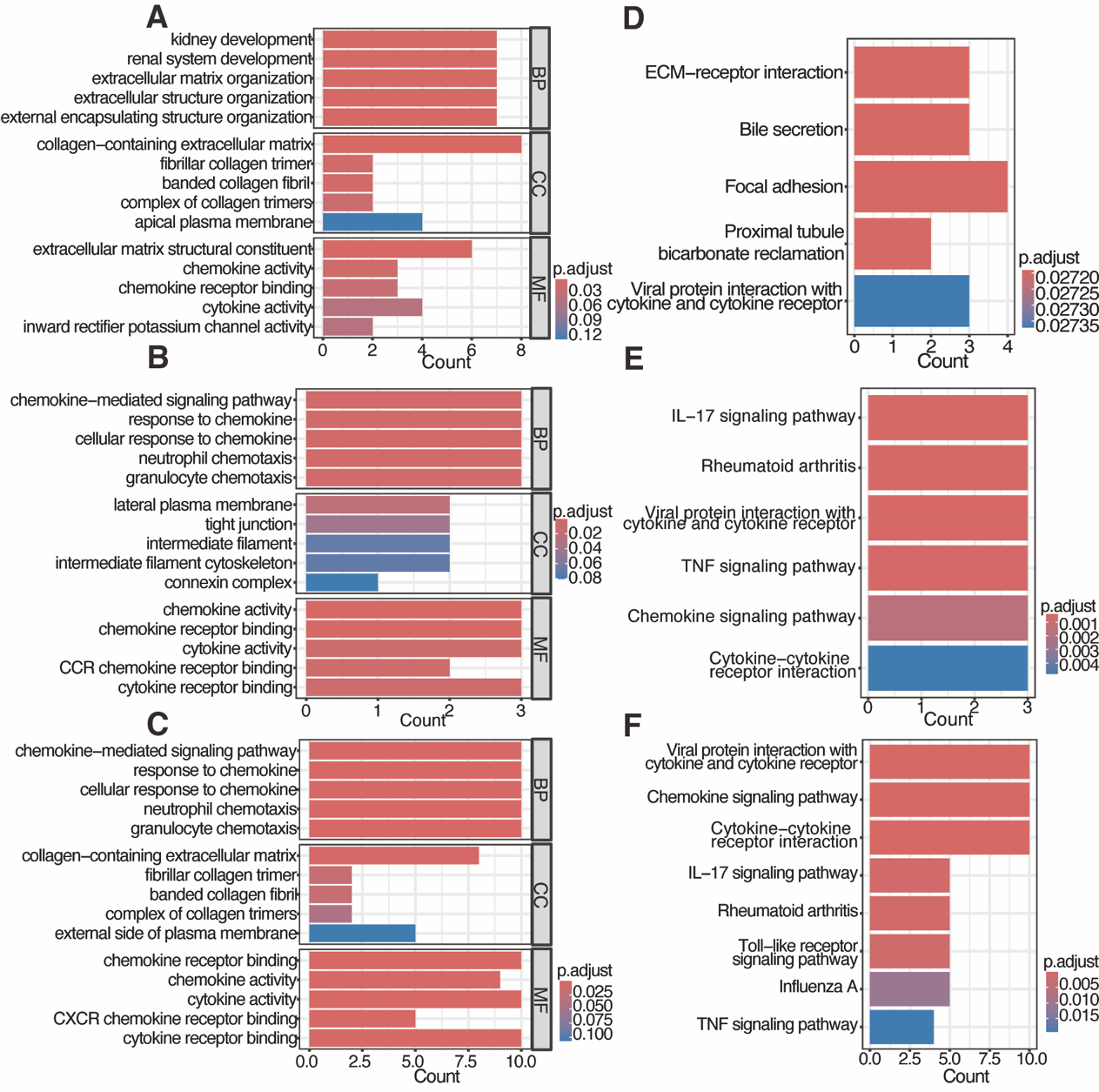


Supplementary Figure 4. GO and KEGG enrichment analysis of the three hub HICGs in LF.

GO enrichment analysis for three hub HICGs: (A) *AQP1*, (B) *GJA1*, and (C) *KCNN2*. (D–F) KEGG pathway enrichment analyses of *AQP1*, *GJA1*, and *KCNN2*. Abbreviations: BP, biological process; CC, cellular component; MF, molecular function.


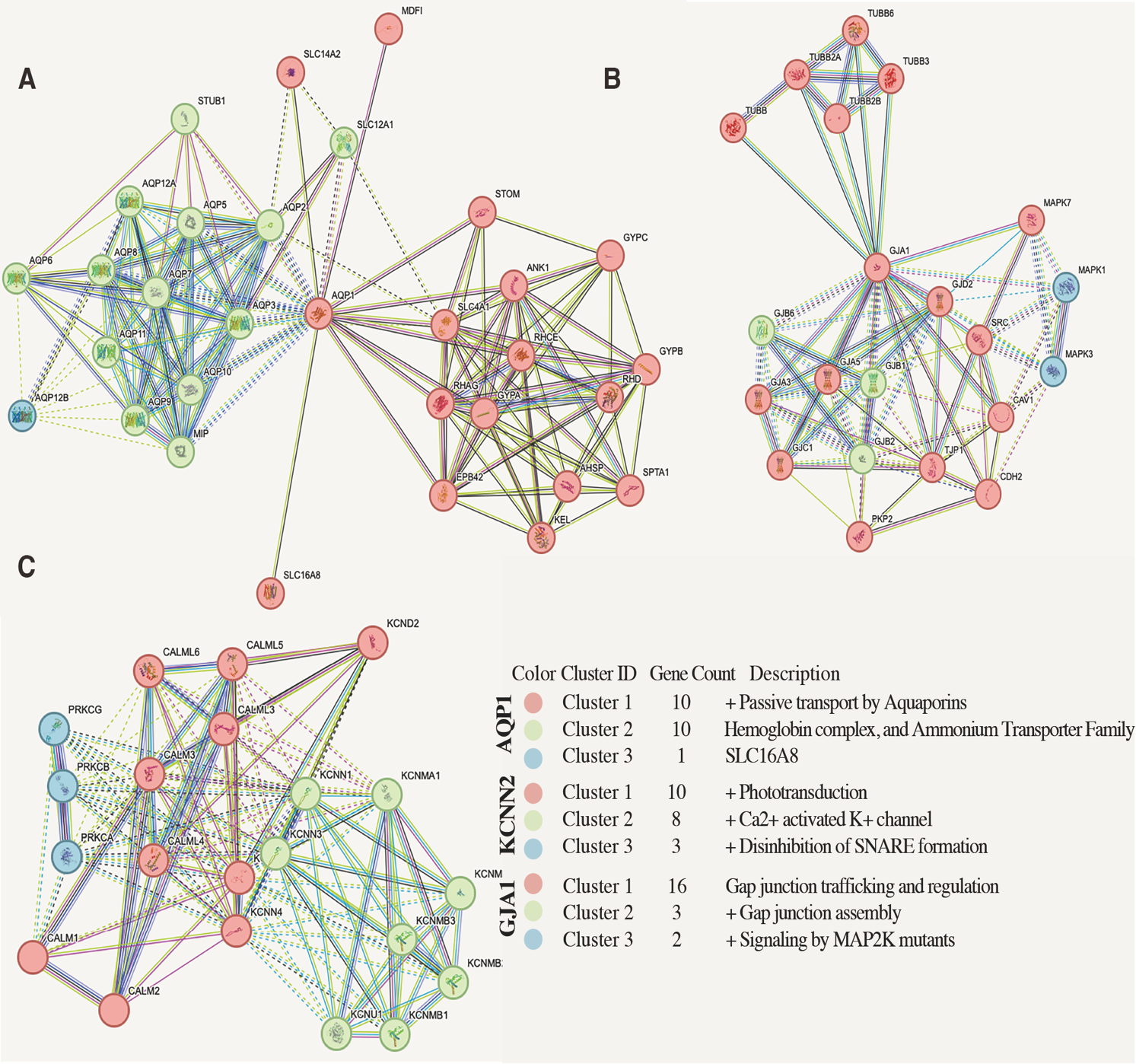


Supplementary Figure 5. STRING-based protein–protein interaction network visualization and functional clustering of HICGs.

(A) AQP1-related network clusters involved in passive water transport and solute carrier pathways; (B) GJA1-centered cluster representing gap junction assembly and trafficking processes; (C) KCNN2-associated cluster enriched in calcium-activated potassium channel activity.


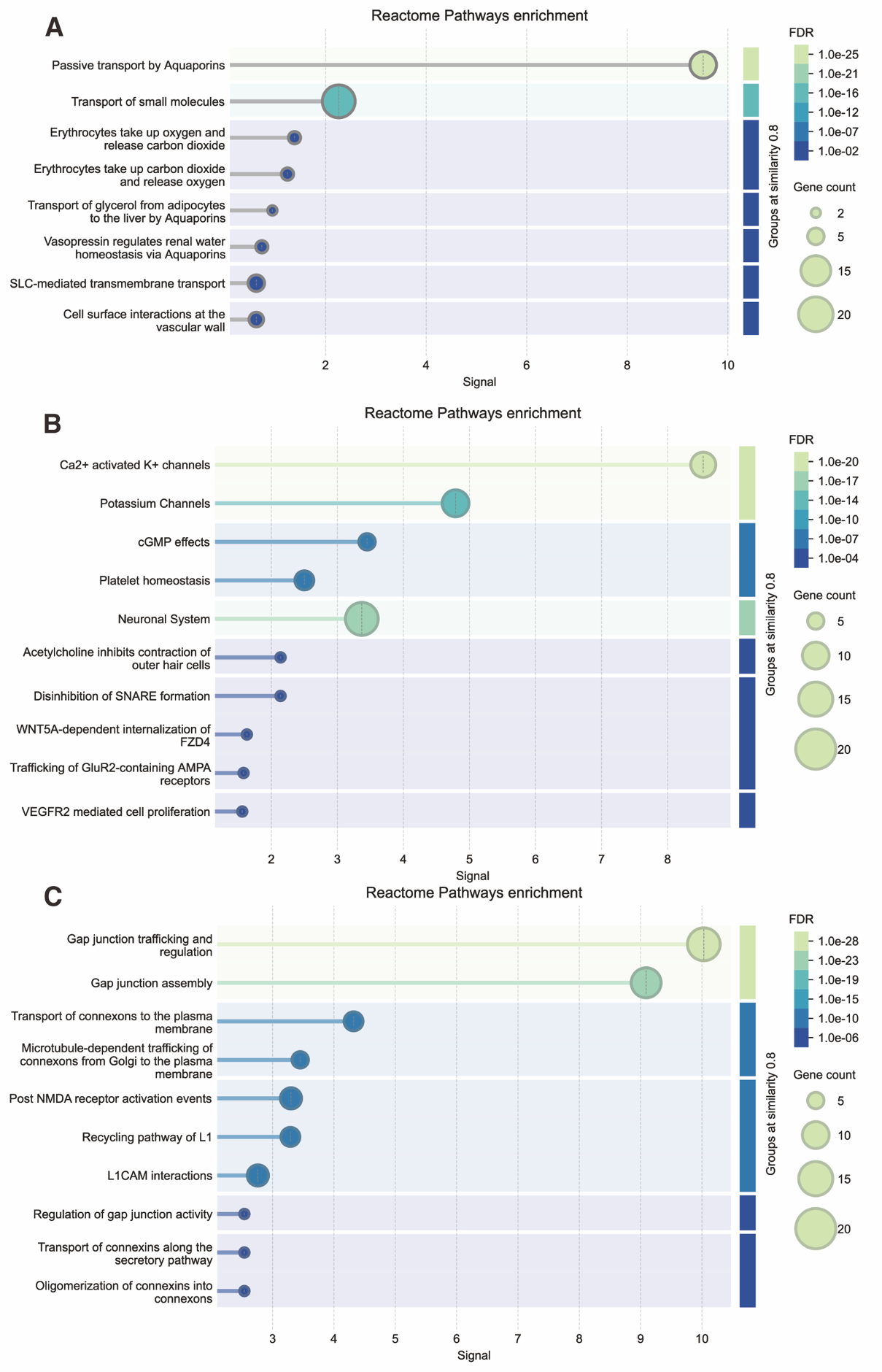


Supplementary Figure 6. Reactome pathway enrichment analysis of PPIs of AQP1(A), KCNN2 (B), and GJA1 (C).


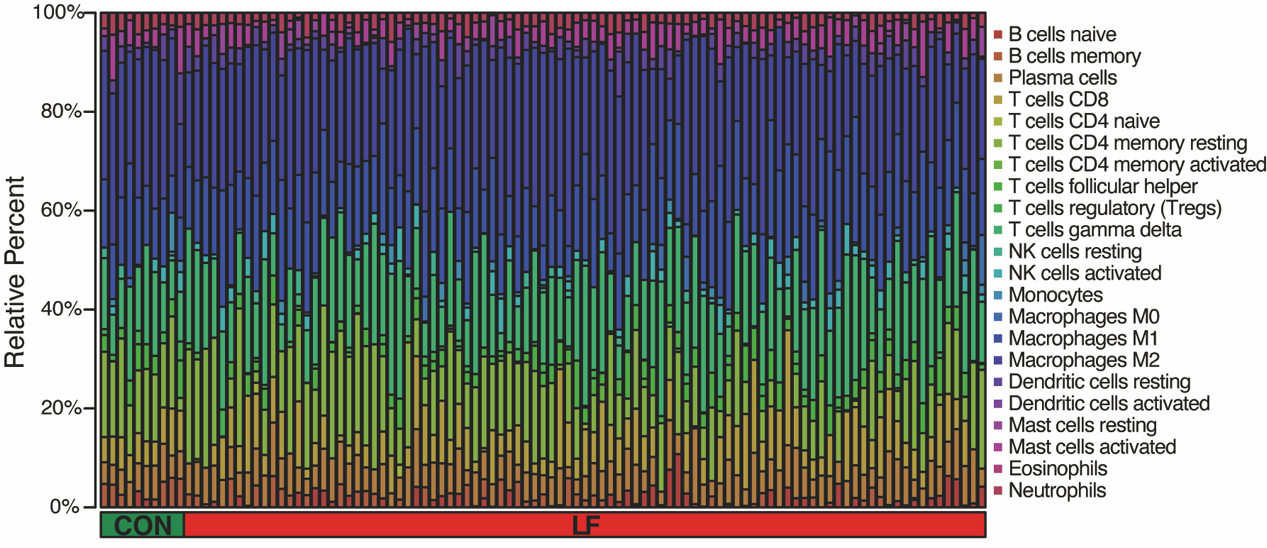


Supplementary Figure 7. Immune cell infiltration landscape in LF and non-fibrosis groups.

Supplementary Table 1. Characteristics of the four included transcriptomic datasets from the GEO database.

| **GEO Accession**​ | **Fibrosis etiology**​ | **Control samples (n)**​ | **Fibrosis samples (n)**​ | **Cohort origin**​ | **Platform**​ |
| --- | --- | --- | --- | --- | --- |
| GSE14323 | Chronic hepatitis C virus | 19 | 41 | United States | GPL96 and GPL571 |
| GSE197112 | Unspecified | 8 | 4 | China | GPL28576 |
| GSE84044 | Hepatitis B virus infection | 43 | 81 | China | GPL570 |
| GSE49541 | Nonalcoholic fatty liver disease | 40 | 32 | United States | GPL570 |

Supplementary Table 2. Diagnostic performance of AQP1, GJA1, and KCNN2 in LF according to different etiologies.

| **Dataset** | **Etiology** | **Gene** | **AUC** | **Cutoff** | **Sensitivity** | **Specificity** |
| --- | --- | --- | --- | --- | --- | --- |
| GSE84044 | Hepatitis B virus infection | *AQP1* | 0.949 (0.898-0.986) | 3.919 | 0.875 | 0.906 |
|  |  | *GJA1* | 0.866 (0.780-0.936) | 6.248 | 0.700 | 0.875 |
|  |  | *KCNN2* | 0.800 (0.685-0.898) | 8.668 | 0.825 | 0.688 |
| GSE49541 | Nonalcoholic fatty liver disease | *AQP1* | 0.949 (0.896-0.987) | 3.919 | 0.875 | 0.906 |
|  |  | *GJA1* | 0.866 (0.773-0.938) | 6.248 | 0.700 | 0.875 |
|  |  | *KCNN2* | 0.800 (0.694-0.902) | 8.668 | 0.825 | 0.688 |
| GSE14323 | Chronic hepatitis C virus | *AQP1* | 0.999 (0.992-1.000) | 7.845 | 0.976 | 1.000 |
|  |  | *GJA1* | 0.991 (0.969-1.000) | 7.690 | 0.902 | 1.000 |
|  |  | *KCNN2* | 0.945 (0.874-0.996) | 4.621 | 0.854 | 1.000 |
| Total | Unspecified | *AQP1* | 0.866 (0.810-0.917) | 6.726 | 0.778 | 0.914 |
|  |  | *GJA1* | 0.796 (0.727-0.856) | 6.611 | 0.635 | 0.829 |
|  |  | *KCNN2* | 0.790 (0.723-0.852) | 7.005 | 0.786 | 0.700 |

Supplementary Table 3: Molecular docking analysis of candidate drugs with their respective targets.

| **Targets** | **Drugs** | **Vina score** | **Cavity volume** | **Contact residues** |
| --- | --- | --- | --- | --- |
| KCNN2 | Alprostadil | -5.8 | 752 | Lys401，Lys404，His405，Asn408，Phe409，Met411，Asp412，Thr416，Gln469，Leu472，Asn473，Asp474，Gln475，Ala476，Asn477，Thr478，Leu479，Val480，Asp481，Lys484 |
|  | Calcitriol | -7.6 | 374 | Gly118，Lys120，Leu121，Gly122，Arg124，Arg125，Phe128，Glu129，Arg131，Lys132，Tyr188，His189，Glu192，Ile193，Leu195，Phe196，Asp199，Asn200，Arg213，Glu220，Ala270，Arg271，Val272，Leu274，Leu275，His276，Ser277，Lys278，Phe280，Thr281，Asp282，Ala283，Ser284，Arg286，Ser287，Ala290，Ile294，Asn295，Phe296，Asn297，Thr298，Val301 |
| GJA1 | Simvastatin | 8.1 | - | Leu7，Leu10，Leu11，Val14，Gln15，Ser18，Gly22，Lys23，Leu26，Ser27，Phe30，Ser86，Thr89，Leu90，Tyr92，Leu93，Tyr155，Ser158，Ile159，Lys162，Asn224，Glu227 |
|  | Rosiglitazone | -6.5 | 4150 | Ala16，Tyr17，Ser18，Thr19，Ala20，Tyr92，Val96，Met100，Glu103，Asn107，Glu110，Lys114，Glu131，Lys134，Phe135，Gly138，Ile139，Glu140，Glu141，His142，Gly143，Lys144，Val145，Lys146，Met147，Arg148，Gly149，Gly150，Leu151，Leu152，Tyr155，Val231，Lys234 |
|  | Raloxifene | -7.8 | 513 | Ile260，Phe263，Leu264，Arg265，Leu266，Tyr267，Ile269，Ala270，Met273，Leu274，Thr298，Arg299，Val301，Met302，Leu305，Thr312，Val313，Leu314，Val316，Phe317，Ile319，Ser320，Leu321，Ile323，Ile324，Ala325，Thr328，Val374，Leu377，Thr378，Met381 |
|  | Propranolol Hydrochloride | -7.6 | 1622 | Gly2，Asp3，Trp4，Ser5，Ala6，Leu7，Gly8，Lys9，Leu10，Leu11，Val14，Ser18，Gly22，Lys23，Leu26，Ser27，Phe30，Ile31，Ile34，Leu35，Leu37，Gly38，Trp78，Val79，Ile82，Ile83，Ser86，Pro88，Thr89，Leu90，Tyr92，Leu93，Tyr155，Ser158，Ile159，Lys162，Asn224，Glu227 |
|  | Valsartan | -6.4 | 1622 | Gly2，Asp3，Trp4，Ser5，Ala6，Leu7，Leu10，Leu11，Val14，Phe30，Ile34，Leu37，Gly38，Val41，Trp78，Val79，Ile82，Ile83，Ser86，Val87，Leu90 |
|  | N-Acetyl-L-Cysteine | -4.5 | 1622 | Leu11，Val14，Ser18，Gly22，Lys23，Leu26，Ser27，Leu29，Phe30，Val85，Ser86，Pro88，Thr89，Leu90，Tyr92，Leu93，Tyr155，Ser158，Ile159，Lys162，Asn224，Glu227 |
|  | Melatonin | -2.5 | - | Met1，Gly2，Trp4，Ile34，Leu37，Gly38，Val41，Glu42 |
|  | Mefloquine | -3.4 | - | Met1，Gly2，Trp4，Ile34，Leu37，Gly38，Val41，Val79 |
|  | Losartan | -7.2 | 93 | Gly2，Asp3，Trp4，Ser5，Ala6，Leu7，Leu10，Leu11，Val14，Phe30，Ile34，Leu37，Gly38，Val41，Trp78，Val79，Ile82，Ile83，Ser86，Val87，Leu90 |
|  | Cerivastatin | -6 | 1622 | Gly2，Asp3，Trp4，Ala6，Leu7，Leu10，Phe30，Ile34，Leu35，Leu37，Gly38，Val41，Val75，Trp78，Val79，Ile82，Ile83，Phe84，Ser86，Leu90 |
|  | Carvedilol | -6.8 | 93 | Asp67，Lys68，Ser69，Phe70，Pro71，Gln173，Trp174，Tyr175，Ile176，Tyr177，Gly178，Phe179，Ser180，Leu181，Ser182，Val184，Tyr185，Thr186，Leu200 |
|  | Calcium D- Pantothenate | -5.1 | 1622 | Trp4，Ser5，Leu7，Gly8，Lys9，Leu11，Asp12，Val14，Gln15，Ser18，Gly22，Lys23，Leu26，Ser27，Leu29，Phe30，Ile31，Arg33，Ile34，Leu35，Val85，Ser86，Pro88，Thr89，Leu90，Tyr92，Leu93，Tyr155，Ser158，Ile159，Lys162，Glu166，Val216，Ser220，Asn224，Glu227 |
|  | Atorvastatin | -7 | 143 | Met147，Arg148，Gly149，Gly150，Leu152，Arg153，Ile156，Ile157，Leu160，Leu228，Phe229，Val231，Phe232，Phe233，Lys234，Gly235，Val236，Asp238，Arg239，Lys241，Gly242，Lys243 |
|  | Ganciclovir | -6.7 | 1622 | Trp4，Leu7，Gly8，Lys9，Leu10，Leu11，Asp12，Val14，Gln15，Tyr17，Ser18，Gly22，Lys23，Val24，Leu26，Ser27，Val28，Leu29，Phe30，Ile31，Arg33，Ile34，Ile82，Val85，Ser86，Val87，Pro88，Thr89，Leu90，Tyr92，Leu93，Tyr155，Ser158，Ile159，Lys162，Glu166，Val216，Ser220，Asn224，Glu227 |
|  | Folic Acid | -7.7 | 1622 | Gly2，Asp3，Trp4，Ser5，Ala6，Leu7，Leu10，Leu11，Val14，Gln15，Ser18，Lys23，Val24，Leu26，Ser27，Phe30，Ile34，Leu37，Gly38，Val41，Glu42，Val75，Val79，Ile82，Ile83，Val85，Ser86，Val87，Thr89，Leu90，Tyr92，Leu93，Tyr155，Ser158，Ile159，Lys162，Asn224，Glu227 |
|  | Diphenhydramine | -7.6 | 1622 | Gly2，Asp3，Trp4，Ser5，Leu7，Gly8，Leu10，Leu11，Asp12，Val14，Gln15，Ser18，Gly22，Lys23，Val24，Leu26，Ser27，Leu29，Phe30，Ile31，Arg33，Ile34，Leu35，Leu37，Val79，Ile82，Ile83，Val85，Ser86，Val87，Thr89，Leu90，Tyr155，Lys162，Glu166，Ser220，Asn224 |
|  | Diazepam | -6.6 | - | Gly2，Asp3，Trp4，Ser5，Leu7，Gly8，Leu10，Leu11，Asp12，Val14，Gln15，Ser18，Gly22，Lys23，Val24，Leu26，Ser27，Leu29，Phe30，Ile31，Arg33，Ile34，Leu35，Leu37，Val79，Ile82，Ile83，Val85，Ser86，Val87，Thr89，Leu90，Tyr92，Leu93，Tyr155，Ser158，Ile159，Lys162，Glu166，Asn224 |
